# Transcriptome-Wide Dynamics of m^6^A Methylation in Tumor Livers Induced by ALV-J Infection in Chickens

**DOI:** 10.3389/fimmu.2022.868892

**Published:** 2022-04-22

**Authors:** Qiqi Zhao, Ziqi Yao, Liyi Chen, Yaai He, Zi Xie, Huanmin Zhang, Wencheng Lin, Feng Chen, Qingmei Xie, Xinheng Zhang

**Affiliations:** ^1^ Heyuan Branch, Guangdong Provincial Laboratory of Lingnan Modern Agricultural Science and Technology & Guangdong Provincial Key Lab of Agro-Animal Genomics and Molecular Breeding & Key Laboratory of Chicken Genetics, Breeding and Reproduction, Ministry of Agriculture, College of Animal Science, South China Agricultural University, Guangzhou, China; ^2^ South China Collaborative Innovation Center for Poultry Disease Control and Product Safety, Guangzhou, China; ^3^ Guangdong Engineering Research Center for Vector Vaccine of Animal Virus, Guangzhou, China; ^4^ United States Department of Agriculture (USDA), Agriculture Research Service, Avian Disease and Oncology Laboratory, East Lansing, MI, United States

**Keywords:** ALV-J, m^6^A, MeRIP-seq, RNA-seq, co-expressed

## Abstract

Avian Leukosis Virus Subgroup J (ALV-J) is a tumorigenic virus with high morbidity and rapid transmission. N6-methyladenosine (m^6^A) is a common epigenetic modification that may be closely related to the pathogenicity of ALV-J. Currently, there are no reports on whether m^6^A modification is related to ALV-J induced tumor formation. In this study, we used methylated RNA immunoprecipitation sequencing (MeRIP-seq) and RNA sequencing (RNA-seq) to examine the differences in m^6^A methylation and gene expression in normal livers and ALV-J-induced tumor livers systematically, with functional enrichment and co-expression analysis. The results identified 6,541 m^6^A methylated peaks, mainly enriched in CDS, and more than 83% of the transcripts contained 1-2 m^6^A peaks. For RNA-seq, 1,896 and 1,757 differentially expressed mRNAs and lncRNAs were identified, respectively. Gene enrichment analysis indicated that they may be involved in biological processes and pathways such as immunology-related and apoptosis. Moreover, we identified 17 lncRNAs, commonly existing in differently expressed methylome and transcriptome. Through co-expression analysis, 126 differentially expressed lncRNAs, and 18 potentially m^6^A-related methyltransferases were finally identified and connected, suggesting that m^6^A modifications might affect gene expression of lncRNAs and play a role in ALV-J induced tumor formation. This study provides the first comprehensive description of the m^6^A expression profile in tumor livers induced by ALV-J infection in chickens, which provides a basis for studying the role of m^6^A modification in ALV-J induced tumorigenesis. This study provides clues for studying the epigenetic etiology and pathogenesis of ALV-J.

## Introduction

Avian Leukosis Virus (ALV) belongs to the Alpharetrovirus genus of the Retrvoviridae family that causes a variety of neoplastic diseases in chickens (1). There are seven subgroups of ALV in chickens, including subgroups A, B, C, D, E, J and K (2). ALV-J is an oncogenic retrovirus that infection can induce erythroblastosis and myelocytomatosis, having the greatest pathogenicity and transmission ability within this class of viruses (3). ALV-J has caused significant economic loss in the poultry industry due to the increased tumors and mortality (4). ALV infection with a concomitant enhanced secondary infection is likely the outcome of immunosuppression (5, 6). ALV-J can be transmitted vertically and horizontally, and horizontal transmission of ALV-J is more efficient than other ALV subgroups (7). ALV-J infection has caused substantial economic losses in the poultry industry worldwide (8–10).

N6-methyladenosine (m^6^A), the methylation modification at the sixth N atom of adenine, is the most common post-transcriptional modification on mRNA, mediating over 60% RNA methylation (11, 12). The modification process of m^6^A RNA is mainly manipulated by three categories of proteins, including “writers”, “erasers” and “reader”, and exert their function by either directly being recognized by m^6^A-binding proteins or readers or indirectly by tuning the structure of the modified RNA to regulate RNA reader-protein interactions (13). RNA modification by m^6^A results in changes in properties such as charge, base pairing, secondary structure and protein-RNA interactions, which in turn affect the transport, localization, translation, and degradation of RNA, ultimately performing the function of regulating gene expression (14, 15). In recent years, many studies demonstrated that m^6^A methylation in mRNA is significantly associated with tumor proliferation, migration, invasion, and metastasis during cancer development and progression (16–18).

LncRNAs are a class of low protein-coding potential RNA with transcripts longer than 200 bp (19). LncRNAs play important roles in transcriptional, post-transcriptional regulation and chromatin modification by regulating gene expression, involving in a variety of biological processes in eukaryotes. Their abnormal expression is closely related to the malignancy of tumors, including tumor proliferation, differentiation, and apoptosis (20). Recent studies have reported that m^6^A-related lncRNAs are associated with the development of tumors and that aberrant lncRNA expression can serve as diagnostic markers in tumors (21). Therefore, exploring the function of lncRNA in ALV-J-induced tumors can help us better understand the pathogenesis of ALV-J.

Up to now, it has not been reported that m^6^A modification is involved in tumor formation induced by ALV-J in chickens. Given the indispensable role of RNA m^6^A modification in various tumors, it is reasonable to surmise that the dysregulation of m^6^A modification might be associated with the tumor formation induced by ALV-J infection in chickens. In this study, we reported the transcriptome-wide m^6^A analysis and the expression of lncRNAs in normal livers and ALV-J induced tumor livers in chickens, and a comprehensive analysis of m^6^A modifications of lncRNAs in ALV-J induced tumor liver tissues. We also analyzed the signaling pathways and potential regulated methyltransferases associated with the m^6^A modification of lncRNAs. These results indicated that m^6^A modification may play an important regulatory role in the tumor formation induced by ALV-J in chickens. We hope that this study will help to further investigate the potential role of lncRNAs with m^6^A modifications in the pathogenesis of ALV-J.

## Materials and Methods

### Animals and Tissue Collection

Specific pathogen-free (SPF) chickens were purchased from Guangdong Wen’s Food Group Co., Ltd. (Yunfu, China), fed in a negative-pressured biosafety isolation chamber with free water and commercial feed. 110 one-day-old SPF chickens were randomly divided into two groups, where each group contains 55 chickens. The first group (positive group, ALV-J induced tumor livers) was inoculated intraperitoneally with 10^3.7^ TCID_50_/0.2 mL ALV-J strain NX0101 at one day of age; the other group (negative group, normal livers) was inoculated with the same volume of nutrient solution, and the negative group served as a control group. 110 days after infection, three chickens in each group were randomly euthanized and necropsied. The liver tissue samples were collected and then quickly frozen in liquid nitrogen and stored at -80°C environment.

The NX0101 strain of ALV-J used in the study was obtained from Professor Cui, Shandong Agricultural University, People’s Republic of China. This experiment strictly adhered to institutional and national guidelines for using and caring of laboratory animals. The use of animals in this study was approved by the South China Agricultural University Committee of Animal Experiments (approval ID: SYXK 2019-0136).

### RNA Extraction and Quality Control

Total RNA of the livers was extracted using TRIzol Reagent (Invitrogen Corporation, CA, USA) according to the manufacturer’s protocols. Three biological replicates were used for positive and negative groups, respectively. The extracted RNA was first detected by PCR for ALV-J infection with the following primers (forward primer: 5′-AGCAACAAGCAAGAAAGACC-3′; reverse primer: 5′-CCGAACCAAAGGTAACACAC-3′). Then, the RNA quantification and quality assurance of each sample were qualified using Qubit 3.0 instrument. RNA integrity and gDNA contamination was identified by denaturing agarose gel electrophoresis.

### MeRIP Sequencing and Analysis

Methylated RNA immunoprecipitation sequencing (MeRIP-seq) was performed on the basis of previously reported methods with some modifications (22). m^6^A RNA immunoprecipitation was performed with the GenSeq™ m^6^A RNA IP Kit (GenSeq Inc., China). Briefly, fragment RNA was incubated with an anti-m^6^A antibody (202003, Synaptic Systems, Germany) in immunoprecipitation buffer at 4°C for 2 hours. The mixture was further immunoprecipitated by incubation using protein A magnetic beads (Thermo Fisher, USA) at 4°C for 2 hours. Then, bound RNA on the magnetic beads was then eluted with N6-methyladenosine (BERRY & ASSOCIATES, PR3732), and then extracted with TRIzol reagent (Thermo Fisher, USA). Purified RNA was constructed by RNA-seq library construction using NEBNext^®^ Ultra II Directional RNA Library Prep Kit (New England Biolabs, Inc., USA). After qualifying with Agilent 2100 Bioanalyzer system (Agilent Technologies Inc., CA, USA), the library sequencing was performed on Illumina NovaSeq 6000 sequencer with 150 bp paired-end.

After sequencing, paired-end reads were generated and quality controlled by Q30, followed by removing adaptors and low-quality reads using the Cutadapt program (v1.9.3) (23). Then, clean reads of input libraries were aligned to the reference genome (GCF_000002315.6_GRCg6a) using STAR, and circRNAs were identified by DCC software (24, 25). After that, clean reads of all libraries were mapped to the reference genome by Hisat2 (v2.0.4) (26). Differential methylation genes and sites were identified using Model-based Analysis of ChIP-Seq (MACS) and diffReps program, respectively (27, 28). These peaks identified by two software overlapping with exon of mRNA, lncRNA and circRNA were screened and annotated. Differentially expressed RNAs were determined by paired t-test algorithm. The thresholds for hyper- or hypomethylation were set as absolute fold change (FC) > 2.0 and P < 0.05. The gene ontology (GO) and Kyoto Encyclopedia of Genes and Genomes (KEGG) pathway enrichment analysis of methylated associated genes using clusterProfiler package in R (29). The GO was categorized as three types: cellular component (CC), biological process (BP), and molecular function (MF). The P value < 0.05 denotes the statistically significant.

### Transcriptome Library Construction and Sequencing

To investigate whether methylated modifications affect gene expression and their molecular mechanisms, we performed transcriptome sequencing for six samples and detected mRNAs, lncRNAs and circRNAs. Briefly, RNA was extracted using the TRIzol method, rRNAs were removed from total RNA using the NEBNext^®^ rRNA Depletion Kit (New England Biolabs, Inc., Massachusetts, USA) following the manufacturer’s instructions, and then, RNA sequencing libraries were constructed using rRNA-depleted RNAs with TruSeq Stranded Total RNA Library Prep Kit (Illumina, USA) after quality control. Library quality control and quantification were performed using the BioAnalyzer 2100 system (Agilent Technologies, USA). Subsequent sequencing of 150 bp paired-end reads on the Illumina Novaseq 6000 sequencer. Raw data was quality controlled by Q30, and the Cutadapt program was used to remove adaptors and low-quality reads. The high-quality reads were aligned to the chicken reference genome (GCF_000002315.6_GRCg6a), and then mRNA, lncRNA and circRNA were detected and identified. Subsequently, differential expression analysis and functional enrichment analysis were performed.

### Co-Express Analysis of m^6^A Methyltransferase and m^6^A-Related lncRNAs

The expression matrixes of 18 m^6^A-related methyltransferases were retrieved from the MeRIP-seq and RNA-seq, including *ALKBH5*, *FTO*, *HNRNPA2B1*, *IGF2BP2*, *IGF2BP3*, *METTL3*, *METTL14*, *METTL16*, *RBM15*, *RBM15B*, *RBMX*, *YTHDC1*, *YTHDC2*, *YTHDF1*, *YTHDF2*, *YTHDF3*, WTAP, and *ZC3H13*. 126 m^6^A-related lncRNAs were identified by Pearson’s correlation analysis. The process used the criteria of |Pearson R| > 0.3 and P < 0.05. The co-expression network was constructed by R, showing it as a Sankey plot.

### Sequencing Data Validation by RT-qPCR and MeRIP-qPCR

Total RNA was extracted from normal livers and ALV-J induced tumor livers using the TRIzol method, and synthesized into cDNA with reverse transcription using PrimeScriptTM RT reagent Kit with gDNA Eraser (Takara, Japan). Then Reverse transcription quantitative real-time PCR (RT-qPCR) was performed using CFX96 Touch (Bio-Rad, USA) following the manufacturer’s instructions. The relative gene expression was normalized to GAPDH, then calculated using 2^−ΔΔCT^ method. The primers using for RT-qPCR were listed in **Table 1**. For the detection of the fold enrichment of m^6^A level, the operation method is similar to RT-qPCR, and the sequences of primers using in MeRIP-qPCR were listed in **Table 2**.

**Table 1:** The primers of lncRNAs for RT-qPCR detection.

LncRNA	Primer sequence	Product size (bp)
*GAPDH* -F	5′-GGGTGGTGCTAAGCGTGTTA-3′	118
*GAPDH* -R	5′-GCACGATGCATTGCTGACAA-3′	
*CMPK2* -F	5′-GTTTCCCGTCGTGGTGTTTG-3′	185
*CMPK2* -R	5′-TGTAGTTGCCCGCAGCATAA-3′	
*HDAC9* -F	5′-CCACTGGGCGGCTATAAAGT-3′	111
*HDAC9* -R	5′-AAGGTCATGTCCTCCCTCCA-3′	
*MINDY4B* -F	5′-GAGGGTACTCACAGGCGATG-3′	156
*MINDY4B* -R	5′-TCCACGTCAGCGTCATTCTC-3′	
*ATOH8* -F	5′-TGCTGCAATCTGAACGGTGT-3′	156
*ATOH8* -R	5′-TCCCCTCTCAGCTCATGTGT-3′	
*LOC100859478* -F	5′-GAGAATGAGCCGTGGTGGAA-3′	87
*LOC100859478* -R	5′-ACCTGACGGTCTCAGGATCA-3′	

**Table 2:** The primers of lncRNAs for MeRIP-qPCR detection.

LncRNA	Primer sequence	Product size (bp)
*GAPDH* -F	5′-GGGTGGTGCTAAGCGTGTTA-3′	118
*GAPDH* -R	5′-GCACGATGCATTGCTGACAA-3′	
*HDAC9* -F	5′-CAACTGAACGTTGCAGGGGA-3′	69
*HDAC9* -R	5′-GCAGAATGCATCCATTATCT-3′	
*MINDY4B* -F	5′-TCCACGTCAGCGTCATTCTC-3′	156
*MINDY4B* -R	5′-GAGGGTACTCACAGGCGATG-3′	
*SOX7* -F	5′-GTACTTCCACCCCAAACGTGA-3′	190
*SOX7* -R	5′-GTTTTCCAGGCAGCATCTCTG-3′	

### Statistical Analysis

Data from three or more independent experiments were presented as the mean ± SEM. Statistical analysis were done using GraphPad Prism 8.0 software. Paired Student’s t-tests were performed between normal livers and ALV-J induced tumor livers. Differences with P < 0.05 were defined as the threshold for significance.

## Results

### Characterization of Normal Livers and ALV-J Induced Tumor Livers

**Figure 1:**
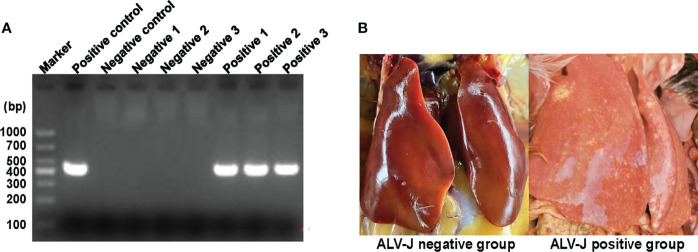
Characterization of normal livers and ALV-J induced tumor livers. **(A)** Electrophoretic analysis showed the infection between the two groups based on the PCR method. **(B)** Tumor liver induced by ALV-J infection in SPF chickens at 110 days. Negative represents chickens without ALV-J infection (normal livers); Positive represents chickens with ALV-J infection (tumor livers).

In order to comprehensively analyze the differences of N6-methyladenosine RNA methylomes between normal livers and ALV-J induced tumor livers in chickens, liver tissues were collected from three randomly selected chickens in positive group (ALV-J induced tumor livers) and negative group (normal livers) at 110 days after infection. PCR results showed that the livers of the infected group were positive for ALV-J, while that of the uninfected group was negative for ALV-J (**Figure 1A**). Compared to the negative group, most chickens in the ALV-J infected group showed gradual emaciation. Tumor livers induced with ALV-J showed hepatomegaly and myeloma symptoms compared with the normal livers in the uninfected group (**Figure 1B**).

### Transcriptome-Wide Detection of m^6^A Methylation in Normal Livers and ALV-J Induced Tumor Livers

MeRIP-seq generated 215,876,754 to 227,827,726 raw reads from IP or input samples from normal livers and ALV-J induced tumor livers. After filtering out low-quality data, more than 96% of high-quality reads from each sample were aligned to the *Gallus* reference genome. Over 86% of the clean reads from all the samples were uniquely mapped to the chicken reference genome (**Supplementary Table S1**). MeRIP-seq analysis of RNA isolated from chicken livers indicated that, 14,584 and 14,057 expressed mRNA transcripts were detected in positive (ALV-J induced tumor livers) and negative groups (normal livers), respectively. After mapping the methylated RNA fragments to the transcriptome, a total of 5,848 m^6^A sites were identified among the 3,597 coding transcripts in the positive group and 6,223 m^6^A sites were identified among 3,637 coding transcripts in the negative group (**Supplementary Table S2**). The proportion of methylated transcripts was 40.10% and 44.27% in positive and negative groups, respectively.

**Figure 2:**
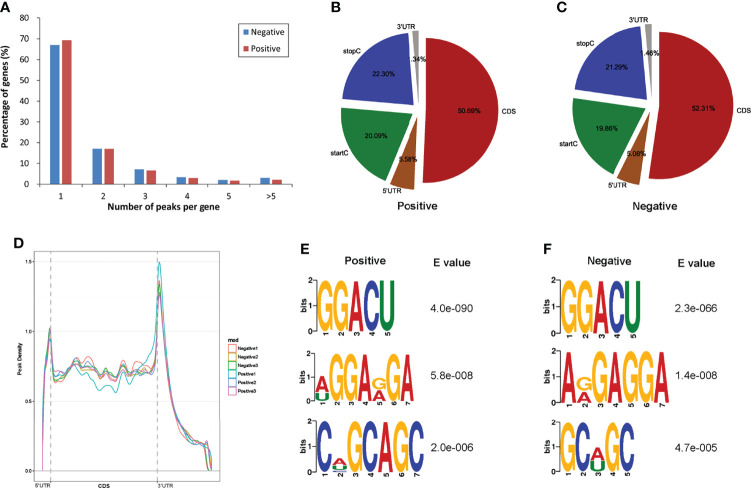
Overview of m^6^A methylation in the normal livers and ALV-J induced tumor livers. **(A)** The proportion of genes harboring different numbers of m^6^A peaks in two groups. **(B, C)** The percentage of m^6^A peaks in five nonoverlapping segments of transcripts in two groups. startC, start codon; CDS, coding sequence; stopC, stop codon; 3’ UTR, the three prime untranslated regions; 5’ UTR, the five prime untranslated regions. **(D)** Accumulation of m^6^A peaks along with transcript in positive and negative groups, respectively. Each transcript is divided into three parts, including 5’ UTR, CDS, and 3’ UTR. **(E, F)** The top three motifs enriched across m^6^A peaks in positive **(E)** and negative group **(F)**. Negative represents chickens without ALV-J infection (normal livers); Positive represents chickens with ALV-J infection (tumor livers).

To determine how the m^6^A modification distributed throughout the chicken transcriptome, we classified the methylated transcripts based on the number of m^6^A peaks contained in each transcript. We found that the number of m^6^A modified sites varies among different transcripts, while more than 83% of modified transcripts contained one or two m^6^A peaks, and about 7.69% of the methylated transcripts contained four or more peaks (**Figure 2A**). In total, the chicken liver transcriptome on average contains 1.71 and 1.61 m^6^A peaks per methylated transcript in positive and normal negative groups, respectively.

To determine the distributed localization of m^6^A in the mRNA transcripts, m^6^A peaks were divided into five non-overlapping segments: 5′UTR, start codon segment (startC), coding sequence (CDS), stop codon segment (stopC) and 3′UTR (**Figures 2B, C**). Our results showed that m^6^A was most commonly present in CDS, with some located near the start and stop codons, and some differences between the two experimental groups, which is consistent with the patterns identified in the mouse, pig and chicken (30–32). The m^6^A peaks near the startC were 20.09% and 19.86% in positive and negative samples, respectively. m^6^A peaks near the CDS reduced 1.62% from negative to the positive group. Furthermore, the abundance of m^6^A peaks near the stopC increased 1.01% in the positive group (ALV-J induced tumor livers). The distribution of m^6^A across the transcriptome was verified by m^6^A reads along the transcripts. Consistent with the distribution of m^6^A peaks, m^6^A reads were distributed throughout the mRNA transcript, where reads were elevated in the CDS and peaked at the 3′UTR. Metagene profiling of the m^6^A peaks showed that they were primarily enriched in CDS, near the start and stop codons, and close to the beginning of 3′UTR and the ending of 5′UTR, which differs from the pattern identified in mammals, and same to chicken (**Figure 2D**) (22, 30). Together, the results reveal that m^6^A is dynamic in ALV-J induced tumor livers in chickens.

Motif enrichment analysis of 1000 peaks within mRNAs with the highest scores (-10×log10(p-value)) obtained from three biological replicates (1,000 peaks per replicate) revealed the conservative RRACH motif (R represents purine, A represents m^6^A, and H represents a nonguanine base), as well as other motifs, in the positive and negative groups, respectively. The results showed that GGACU was significantly enriched and consistently considered to be the beat motif in both groups, suggesting that the RRACH motif employed in the genesis of ALV-J is conserved in the presence or absence of ALV-J infection, which means that GGACU is a commonly present m^6^A modification sequence (**Figures 2E, F**).

### Distribution of Differentially Methylated m^6^A Sites in Normal Livers and ALV-J Induced Tumor Livers

**Figure 3:**
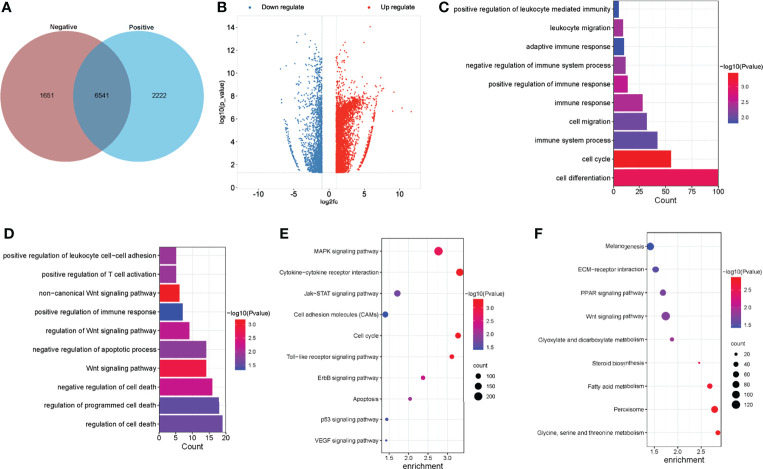
Global m^6^A modification changes in the normal livers and ALV-J induced tumor livers. **(A)** Venn diagram of m^6^A methylation sites identified in mRNAs from two groups. **(B)** The Volcano plot showed the identification of significant hyper- and hypomethylated m^6^A peaks. **(C, D)** GO enrichment analysis of biological processes involved in hypermethylated **(C)** and hypomethylated **(D)** m^6^A genes in ALV-J infected positive group compared with negative group. **(E, F)** Pathway analysis of hypermethylated **(E)** and hypomethylated **(F)** m^6^A genes in mRNAs of ALV-J infected positive group compared to negative group. Negative represents chickens without ALV-J infection (normal livers); Positive represents chickens with ALV-J infection (tumor livers).

To investigate the differences in m^6^A modifications between the positive group (ALV-J induced tumor livers) and negative group (normal livers), we initially identified m^6^A peaks and genes specific to both groups. We found 6,541 peaks that were methylated in both positive and negative groups, and 2,222 and 1,651 peaks that were specifically hypermethylated and hypomethylated in the mRNA of positive against negative group, respectively (**Figure 3A**). Of which, 68.48% (4,858/7,094) were significantly hypermethylation among the differentially expressed methylation sites (positive vs. negative, **Figure 3B**). Similarly, we screened 99 methylation peaks in lncRNAs that were present simultaneously in two groups, and 67 specific hypermethylated peaks and 50 specific hypomethylated peaks were identified in positive group against negative group (**Supplementary Figure S1**). We also identified 561 differentially methylated m^6^A sites within 172 lncRNA transcripts, of which 71.30% (400/561) were hypermethylation sites (positive vs. negative). A total of 138 differentially methylated m^6^A sites within 95 circRNA genes, of which 73.19% (101/138) were hypermethylated (positive vs. negative). **Table 3** showed the top 20 hyper- and hypomethylated m^6^A sites within mRNAs with the highest values of fold change, of which were >65-fold.

**Table 3:** The top 20 differently methylated m^6^A peaks (positive vs. negative).

Gene Name	Gene ID	Fold change	P value	Peak length	Regulation
*BLB3*	425256	3203.26423	2.3119E-07	280	up
*LOC107051636*	107051636	1306.59562	1.141E-07	379	up
*CCLI8*	771679	583.5	4.6837E-09	109	up
*MR1*	100859628	513.732692	2.1187E-07	49	up
*CENPF*	395357	210.9	2.1556E-09	149	up
*HIST1H2A4L1*	100858459	185	2.3954E-09	219	up
*LOC107052493*	107052493	176.1	3.5396E-09	179	up
*SIK1*	395417	160.9	2.5641E-10	239	up
*DYNC1I1*	420571	149.8	1.2503E-09	118	up
*TPM3*	770103	126.4	4.6012E-09	161	up
*KCTD16*	427644	117.2	4.3087E-11	393	down
*TRIM29*	419754	107.7	2.0086E-08	153	down
*PEX11G*	420131	105.7	3.8967E-08	106	down
*LOC107055024*	107055024	82	1.182E-06	379	down
*MYH1G*	427789	74.2	2.5322E-06	171	down
*CCDC170*	421639	72.8	6.0886E-06	211	down
*SGIP1*	776038	71.7	6.2746E-06	80	down
*LOC107055318*	107055318	70.8	1.605E-06	114	down
*MYH1C*	417310	70.3	9.2969E-06	171	down
*CLCN5*	422285	67.46	2.2999E-11	130	down

m^6^A, N6-methyladenosine.

### Differentially Methylated mRNAs Involved in Important Biological Pathways in ALV-J Induced Tumor Livers

To reveal the function of m^6^A in ALV-J induced tumor livers in chickens, protein-coding genes containing differentially methylated m^6^A sites were collected for GO enrichment and KEGG pathway analysis. As for the BP category of mRNA, genes with hypermethylated m^6^A sites were significantly enriched in immune related terms (13/193) such as regulation of immune response/process, immune system development, adaptive immune response, while genes with hypomethylated m^6^A sites were highly involved in regulation of cell death/apoptosis in ALV-J induced tumor livers (7/86, P < 0.05, **Figures 3C, D**). For the CC category, hypermethylated m^6^A genes were mainly enriched in membrane and chromosome. For the MF category, hypermethylation of m^6^A in the positive group was notably enriched in transferase activity, kinase regulator activity (**Supplementary Table S3**). Compared with the negative group of chickens which were not infected by ALV-J, hypermethylation in tumor livers of chickens were more involved in regulating the immune system, while hypomethylation in tumor livers of chickens were more participated in cell apoptosis. Furthermore, genes with hypermethylated m^6^A sites were found to be significant in associated pathways, such as Toll-like receptor, MAPK, apoptosis, VEGF, and JAK-STAT signaling pathway, while hypomethylated m^6^A sites were highly related to PPAR and Wnt signaling pathway (P < 0.05, **Figures 3E, F**).

### Overview of RNA Expression Profiles in Normal Livers and ALV-J Induced Tumor Livers

**Figure 4:**
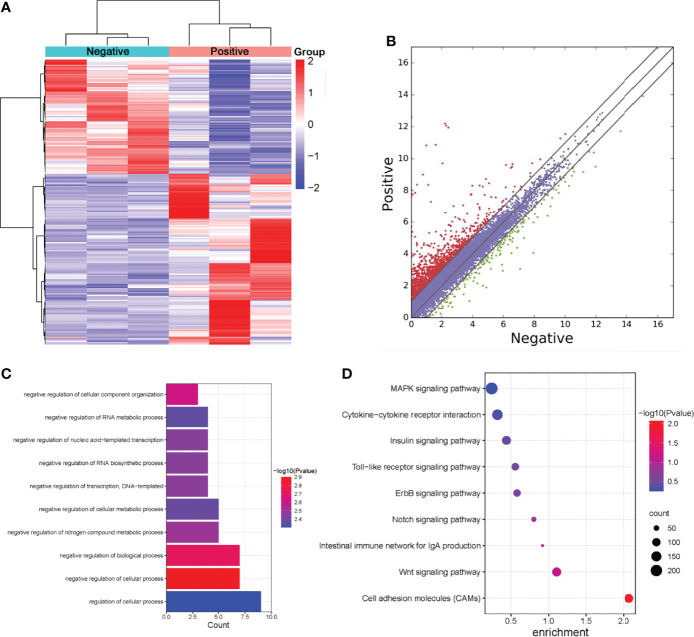
Screening of differentially expressed genes in normal livers and ALV-J induced tumor livers by RNA-seq. **(A)** Heatmap of RNA-seq data in two groups. Rows: mRNAs; columns: ALV-J infected positive and negative groups. Red, black, and green indicate the up-regulation, unchanged, and down-regulation of mRNAs, respectively. **(B)** Differentially expressed mRNAs in ALV-J infected positive and negative groups. Red dots represent up-regulated genes in the positive group compared with the negative group, green dots represent down-regulated genes, and blue dots represent genes with no significant differences. **(C)** GO enrichment of biological processes involved in up-regulated genes in the positive group compared to the negative group. **(D)** Pathway analysis of up-regulated genes in the positive group. Negative represents chickens without ALV-J infection (normal livers); Positive represents chickens with ALV-J infection (tumor livers).

To understand whether m^6^A modifications could affect gene expression in ALV-J induced tumor livers, RNA-seq data were firstly used to investigate the differential expression of genes between the two groups. The expression patterns of RNA were investigated by RNA-seq, from which the expression level of 15,363 mRNAs, 31,279 lncRNAs, and 4,705 circRNAs were found in the six samples, respectively. Differently expressed genes between the positive (ALV-J induce tumor livers) and negative (normal livers) groups were further screened. Compared with the negative group, 1,896 mRNAs, 339 lncRNAs, and 43 circRNAs with differential expression in ALV-J induced tumor livers were obtained, including 1,687 up-regulated mRNAs, 295 up-regulated lncRNAs, and 29 up-regulated circRNAs, respectively. The hierarchical clustering and scatter plot of the RNA-seq data were shown in **Figures 4A, B**, indicating that these RNAs have different expression patterns in two groups.

To further understand the effects of lncRNAs, we annotated the differentially expressed lncRNAs and analyzed their GO enrichment and KEGG pathways. GO analysis revealed that positive up-regulated genes in ALV-J induced tumor livers were significantly enriched in biological processes involving regulation of the cellular process, regulation of cellular metabolic process, and negative regulation of RNA biosynthetic process (**Figure 4C**). Moreover, KEGG analysis showed that MAPK, cytokine-cytokine receptor interaction, Toll-like receptor, the intestinal immune network for IgA production, and Wnt pathways were significantly altered in up-regulated lncRNAs (**Figure 4D**). Together, these results indicated that m^6^A modification might influence RNA expression, thereby regulating the tumor formation induced by ALV-J infection.

### Screening the Common Differential Genes Between lncRNAs Methylation and Gene Expression of lncRNAs in ALV-J Induced Tumor Livers

**Figure 5:**
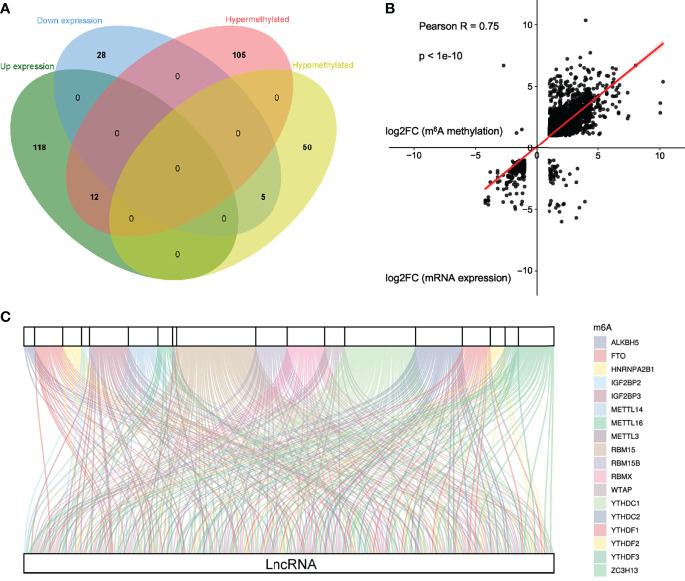
Conjoint analysis of m^6^A methyltransferases and lncRNA genes in ALV-J induced tumor livers. **(A)** Venn diagram showing the relationship between m^6^A methylation and lncRNA expression level. **(B)** Positive correlation between m^6^A methylation and mRNA expression level (Pearson R=0.75; p<1e-10). **(C)** Sankey relational diagram for 18 m^6^A genes and 126 m^6^A-related lncRNAs.

To obtain differentially expressed lncRNAs shared in MeRIP and RNA-seq, a few lncRNAs with both differential methylation and expression levels in ALV-J-induced tumor livers were identified in two sequencing results. Thus, 17 differentially m^6^A-methylated lncRNAs were screened (**Figure 5A**). Besides, their associated genes were found, including *HDAC9*, *CMPK2*, *MINDY4B*, *ATOH8*, *SOX7*, *LOC100859478*, etc. Among them, five lncRNAs (*LOC107052235*, *ATOH8*, *LOC101751658*, *SOX7*, and *LOC100859478*) were hypomethylated and low expressed in ALV-J induced tumor livers, while the other lncRNAs like *HDAC9*, *CMPK2*, *MINDY4B*, etc., was hypermethylated and highly expressed in ALV-J induced tumor livers. The details were shown in **Table 4**.

**Table 4:** Common differential genes in MeRIP-seq and RNA-seq.

Transcript id	Peak length	P value	Regulation	Gene name	Location
XLOC_003562	68	1.33024E-08	up & hyper	*HDAC9*	intron sense-overlapping
XLOC_006040	17	1.26006E-06	up & hyper	―	intergenic
XLOC_007199	282	4.89894E-07	up & hyper	*CMPK2*	exon sense-overlapping
XLOC_017992	239	4.69617E-07	up & hyper	―	intergenic
XLOC_024250	910	5.69015E-06	up & hyper	―	intergenic
LOC112531149	199	1.46458E-06	up & hyper	―	intergenic
LOC101751016	105	6.27978E-08	up & hyper	―	intergenic
LOC112531349	996	2.90995E-06	up & hyper	―	intergenic
LOC107052842	339	6.78792E-08	up & hyper	―	intergenic
LOC112532710	199	4.56211E-06	up & hyper	―	intergenic
LOC107054147	559	2.23371E-06	up & hyper	*MINDY4B*	natural antisense
LOC107054686	419	4.92186E-06	up & hyper	―	intergenic
LOC107052235	239	7.71317E-13	dn & hypo	―	intergenic
LOC107053315	162	2.75528E-13	dn & hypo	*ATOH8*	natural antisense
LOC101751658	64	2.33973E-06	dn & hypo	―	intergenic
LOC112532116	329	7.1866E-07	dn & hypo	*SOX7*	natural antisense
LOC100859478	754	5.96176E-08	dn & hypo	*LOC100859478*	exon sense-overlapping

up & hyper, upregulated and hypermethylated genes; dn & hypo, down-regulated and hypomethylated genes.

### Co-Expression Analysis of m^6^A Methyltransferases and lncRNAs in ALV-J Induced Tumor Livers

By cross-analysis of the MeRIP-seq and RNA-seq data, we discovered a positive correlation of differentially methylated m^6^A sites and gene expression levels in normal livers and ALV-J induced tumor livers (**Figure 5B**). To explore the potential target lncRNAs associated with differentially expressed m^6^A methyltransferases in ALV-J induced tumor livers, we analyzed the expression correlation of all differentially expressed mRNAs with them. According to the previous studies (33), the expression matrixes of 18 m^6^A methyltransferases were obtained from the MeRIP-seq and RNA-seq data, including the expression of writers *METTL3*, *METTL14*, *METTL16*, *RBM15*, *RBM15B*, *ZC3H13*, and *WTAP*), readers (*IGF2BP2*, *IGF2BP3*, *YTHDC1*, *YTHDC2*, *YTHDF1*, *YTHDF2*, *YTHDF3*, *HNRNPA2B1*, and *RBMX*), and erasers (*ALKBH5*, and *FTO*). After integrating these genes, a co-expression network was constructed for 18 m^6^A methyltransferases and 126 differentially expressed genes of lncRNAs to visually manifest the relationship (**Figure 5C**, **Supplementary Table S4**). From the Sankey diagram, it was clear that *RBM15*, *YTHDC1*, and *YTHDC2* were associated with more lncRNAs ― over 37% (147/397) ― indicating that these methylation-related enzymes have important catalytic and recognition roles in the occurrence of the tumor formation induced by ALV-J infection. Likewise, we detected expressed genes *LOC112530919*, *RAD50*, *LOC107054512*, *FBXO40*, *ARGLU1*, *ELOC*, *RBM39*, *KHDRBS1*, and so on, that were connected with more methylation signals, involved in the methylation process of m^6^A modifications as major factors during disease onset and progression. Together, m^6^A modification can regulate the function of lncRNAs through the binding of reader proteins, which can affect the progression of tumor formation.

### Differentially Expressed lncRNAs and m^6^A Modification Levels of lncRNAs Were Confirmed by RT-qPCR and MeRIP-qPCR

**Figure 6:**
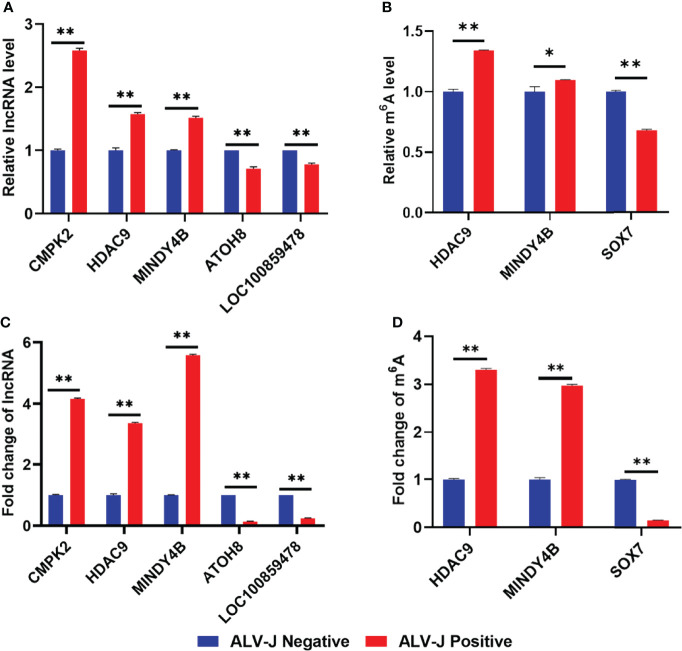
Validation of differentially expressed lncRNAs and m^6^A modification levels of lncRNAs in normal livers and ALV-J induced tumor livers using RT-qPCR and MeRIP-qPCR. **(A)** The relative lncRNA expression level of *CMPK2*, *HDAC9*, *MINDY4B*, *ATOH8*, and *LOC100859478* in ALV-J induced tumor livers (positive) and normal livers (negative) was detected by RT-qPCR. **(B)** The m^6^A level of *HDAC9*, *MINDY4B*, and *SOX7* was validated by MeRIP-qPCR. Expression levels were normalized by *GAPDH*. **(C)** The lncRNA fold change of *CMPK2*, *HDAC9*, *MINDY4B*, *ATOH8*, and *LOC100859478* in ALV-J induced tumor livers (positive) and normal livers (negative) in RNA-Seq results**. (D)** The m^6^A level of *HDAC9*, *MINDY4B*, and *SOX7* in MeRIP-seq results. Data are presented as mean ± SEM. **P < 0.05; *P < 0.01. Negative represents chickens without ALV-J infection (normal livers); Positive represents chickens with ALV-J infection (tumor livers).

To confirm the accuracy of RNA-seq, five lncRNAs were selected and confirmed by RT-qPCR. RT-qPCR analysis revealed that the lncRNA levels of *HDAC9*, *CMPK2*, *MINDY4B* were up-regulated in the ALV-J induced tumor livers (positive group) compared to the normal livers in the negative group, whereas lncRNA levels of *ATOH8* and *LOC100859478* were down-regulated in the positive group (**Figure 6A**). Furthermore, to confirm the accuracy of MeRIP-seq, we selected three transcripts (*HDAC9*, *MINDY4B*, *SOX7*) and the m^6^A modification in these three transcripts were further verified by MeRIP-qPCR. MeRIP-qPCR results showed that the relative level of m^6^A modification in *HDAC9*, *MINDY4B* was significantly increased in ALV-J induced tumor livers, while the relative level of m^6^A modification of *SOX7* in ALV-J induced tumor livers was significantly decreased (**Figure 6B**). These results of RT-qPCR and MeRIP-qPCR were consistent with the data of RNA-seq (**Figure 6C**) and MeRIP-seq (**Figure 6D**), indicating that the sequencing data and the analysis of this study are credible and valid. Thus, these data suggested that m^6^A mediates dynamic gene expression of ALV-J induced tumor formation.

## Discussion

ALV is a highly infectious and oncogenic disease that threatens poultry farming countries worldwide (34). Like HIV, which belongs to the same retrovirus, different subtypes of ALV have different clinical features, and the pathogenesis of ALV-J is particularly complex (35, 36). Epigenetic changes associated with RNA reversible chemical modifications play an essential role in the life cycle of the virus. A growing number of evidence discusses that m^6^A modifications are closely associated with the development and progression of different cancers, anticipating the use of m^6^A methylation as a new cancer diagnostic biomarker and therapeutic target (33, 37). Han et al. discovered that *METTL3* affects the proliferation of bladder cancer by regulating m^6^A modifications in non-coding RNAs (38). It was found that dynamic changes in m^6^A of lncRNA play a vital regulatory role in chicken Marek’s disease virus replication by constructing a transcriptome-wide m^6^A modification map of Marek’s disease (19). With these examples, we suspected that m^6^A modifications also contribute to tumor formation in ALV-J infected chickens by affecting gene expression. Here, we report that the dynamic presence of m^6^A modifications in chicken liver transcripts with and without ALV-J infection affects gene expression.

Infection with ALV-J leads to dynamic changes in m^6^A and accumulation in tumor liver tissues, providing evidence for disease-induced changes in m^6^A status. In this study, a chicken liver model with or without ALV-J infection was established to assess m^6^A modification and revealed its differences in the tumor livers of chickens after ALV-J infection, supporting the dynamic characterization of m^6^A modifications. To our knowledge, this is the first report about the high-throughput study of RNA methylation in liver tissues of chickens infected with ALV-J. The data showed that extensive mRNA hypermethylation and hypomethylation occurred in liver tissues during the occurrence of tumor formation induced by ALV-J infection, further suggesting that m^6^A modification may influence ALV-J induced tumor formation by regulating gene expression.

LncRNA is a type of transcript with a length of more than 200 nucleotides and has no protein-coding function, which significantly impacts chromatin organization, transcription, and post-transcriptional regulation (39, 40). To investigate the effect of m^6^A modification in lncRNA on tumor formation induced by ALV-J infection, our results showed an increase in m^6^A modification of lncRNA in ALV-J induced tumor livers. The m^6^A modifications were positively correlated with mRNA expression in the tumor liver samples, and we further confirmed that there was a close relationship between lncRNA and m^6^A modification in the formation of tumor livers induced by ALV-J infection. m^6^A is prevalent in mRNA transcripts from chicken liver tissue, with m^6^A reaching its highest value near stop codon and then decreasing at the 3′UTR. This distribution pattern is consistent with previous findings in mice (31). GO and KEGG analysis identified the differential expression of m^6^A and lncRNAs associated with specific signaling pathways in ALV-J induced tumor livers. This study predicted the Toll-like, MAPK, cytokine-cytokine receptor interaction, ErbB pathways (ALV-J induced tumor livers vs. normal livers) and found that lncRNAs with m^6^A modifications were common in tumor livers induced by ALV-J infection; hence, we suspected that lncRNAs with m^6^A modifications might mediate tumor formation and mediate viral immunosuppression. It was reported that high expression of Toll-like receptors contributes to epithelial ovarian carcinogenesis, inhibits the development of bladder cancer, and serves as a potential therapeutic target for colorectal cancer (41, 42). Similarly, the ErbB signaling pathway regulates human malignancies like breast, lung, and bladder cancer, allowing for cancer prevention and treatment (43–45). Nevertheless, m^6^A differences were investigated to reveal potential effects. However, further analysis is still needed to validate these results. These suggest that m^6^A-modified lncRNAs affect the occurrence and development of tumor formation inducted by ALV-J infection through biological processes, cellular composition, molecular functions, and signaling pathways.

According to the correlation analysis of MeRIP-seq and RNA-seq, we identified 17 m^6^A-modified lncRNAs, most of which are present in the intergenic region, which is very similar to the definition of lncRNAs that cannot encode proteins but may cause functional changes in mRNAs through structural changes (40, 46). Furthermore, we performed co-expression network construction using 18 methyltransferases and 126 lncRNAs. We identified the crucial functional factor and key lncRNAs, which will provide a theoretical basis for future studies on m^6^A modification of lncRNA to induce tumor formation induced by ALV-J infection in chickens. Regulation of m^6^A modifications may become a strategy for treating the disease induced by ALV-J in chickens in the future. Currently, there are no reports of m^6^A modification of lncRNAs on HIV. We trust that the results of this study may provide new insights into the pathogenesis of HIV, especially that m^6^A modification of lncRNAs may be a factor affecting HIV pathogenesis and explore possible prevention and control methods.

Overall, this study used transcriptomic and m^6^A methylationome data to integrally analyze differential lncRNA with m^6^A modifications in ALV-J-induced tumor livers. Comparing ALV-J-induced tumor livers with normal livers, we identified a few functional lncRNAs and methyltransferases, revealing a tight relationship between m^6^A methylation and chicken tumor formation induced by ALV-J infection. Therefore, the results of this study provide a new vision and theoretical basis for the prevention and control of subgroup J avian leukosis.

## Data Availability Statement

The datasets presented in this study can be found in online repositories. The names of the repository/repositories and accession number(s) can be found below: https://www.ncbi.nlm.nih.gov/, GSE192892.

## Ethics Statement

This experiment strictly adhered to institutional and national guidelines for using and caring of laboratory animals. The use of animals in this study was approved by the South China Agricultural University Committee of Animal Experiments (approval ID: SYXK 2019-0136).

## Author Contributions

QX and XZ designed the study. QZ and XZ wrote the manuscript. ZY, LC, YH, and ZX performed the experiments. QZ and ZY analyzed the results. HZ, WL, FC, and QX supervised the project and critically revised the manuscript. All authors contributed to the article and approved the submitted version.

## Funding

This work was supported by the Key Research and Development Program of Guangdong Province (2020B020222001), the National Natural Science Foundation of China (Grant no. 31902252, 31972659), Guangdong Basic and Applied Basic Research Foundation (2019A1515012006), Chief expert Project of Agricultural Industry Technology system in Guangdong Province (2019KJ128), and Special Project of National Modern Agricultural Industrial Technology System (Grant no. CARS-41).

## Conflict of Interest

The authors declare that the research was conducted in the absence of any commercial or financial relationships that could be construed as a potential conflict of interest.

## Publisher’s Note

All claims expressed in this article are solely those of the authors and do not necessarily represent those of their affiliated organizations, or those of the publisher, the editors and the reviewers. Any product that may be evaluated in this article, or claim that may be made by its manufacturer, is not guaranteed or endorsed by the publisher.
