# Supplementary material for: Transcriptome-Wide Dynamics of m6A Methylation in Tumor Livers Induced by ALV-J Infection in Chickens
Source: Front Immunol. 2022 Apr 22;13:868892. doi: 10.3389/fimmu.2022.868892 (PMC9072629; doi:10.3389/fimmu.2022.868892)
Supplement: Supplementary file 1 [file DataSheet_1.docx]

***Supplementary Material***

**Supplementary Figures**

**
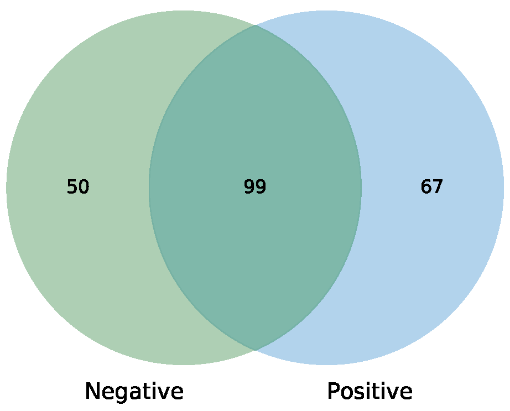
**

**Supplementary Figure S1 |** Venn diagram of m^6^A methylation sites identified in lncRNAs from two groups.

**Supplementary Tables**

**Supplementary Table S1 |** Summary of sequencing data and reads alignment statistics from MeRIP-seq in ALV-J induced tumor livers and normal livers.

| **Sample** | **Raw reads** | **Clean reads** | **Mapped reads** | **Mapped ratio (%)** |
| --- | --- | --- | --- | --- |
| Positive-IP | 217,607,088 | 212,276,658 | 184,322,101 | 86.83% |
| Positive-Input | 227,827,726 | 227,790,522 | 213,339,016 | 93.64% |
| Negative-IP | 215,876,754 | 212,862,030 | 187,183,559 | 87.91% |
| Negative-Input | 223,146,596 | 223,106,230 | 211,074,398 | 94.60% |
| Note: Negative, normal livers; Positive, ALV-J induced tumor livers. | | | | |

**Supplementary Table S2 |** Statistics of m^6^A sites and coding transcripts in ALV-J induced tumor livers and normal livers.

| **Sample** | **N1** | **N2** | **N3** | **P1** | **P2** | **P3** |
| --- | --- | --- | --- | --- | --- | --- |
| Number of m^6^A sites | 5,751 | 6,950 | 5,968 | 4,119 | 6,455 | 6,969 |
| Number of coding transcripts | 3,435 | 3,978 | 3,498 | 2,841 | 3,777 | 4,173 |
| Note: N, Negative sample (normal livers); P, Positive sample (ALV-J induced tumor livers); m^6^A, N6-methyladenosine. | | | | | | |

**Supplementary Table S3 |** GO analysis of hypermethylated m^6^A sites in ALV-J induced tumor livers.

| **GO ID** | **Description** | **Ontology** | **Count** | **P value** | **Gene Ratio** |
| --- | --- | --- | --- | --- | --- |
| GO:0000793 | condensed chromosome | CC | 13 | 7.54E-05 | 3.67E-02 |
| GO:0000776 | kinetochore | CC | 11 | 7.13E-04 | 3.11E-02 |
| GO:0071944 | cell periphery | CC | 101 | 8.55E-04 | 2.85E-01 |
| GO:0005886 | plasma membrane | CC | 97 | 1.40E-03 | 2.74E-01 |
| GO:0005694 | chromosome | CC | 43 | 1.83E-03 | 1.21E-01 |
| GO:0005856 | cytoskeleton | CC | 57 | 2.03E-03 | 1.61E-01 |
| GO:0000777 | condensed chromosome kinetochore | CC | 8 | 3.20E-03 | 2.26E-02 |
| GO:0043228 | non-membrane-bounded organelle | CC | 107 | 3.31E-03 | 3.02E-01 |
| GO:0043232 | intracellular non-membrane-bounded organelle | CC | 107 | 3.31E-03 | 3.02E-01 |
| GO:0019898 | extrinsic component of membrane | CC | 13 | 3.40E-03 | 3.67E-02 |
| GO:0016757 | transferase activity, transferring glycosyl groups | MF | 14 | 2.92E-04 | 4.38E-02 |
| GO:0140097 | catalytic activity, acting on DNA | MF | 15 | 7.66E-03 | 4.69E-02 |
| GO:0008092 | cytoskeletal protein binding | MF | 35 | 7.91E-03 | 1.09E-01 |
| GO:0005096 | GTPase activator activity | MF | 8 | 8.15E-03 | 2.50E-02 |
| GO:0019207 | kinase regulator activity | MF | 11 | 9.15E-03 | 3.44E-02 |
| GO:0003677 | DNA binding | MF | 75 | 1.27E-02 | 2.34E-01 |
| GO:0042802 | identical protein binding | MF | 36 | 2.29E-02 | 1.13E-01 |
| GO:0004527 | exonuclease activity | MF | 5 | 2.53E-02 | 1.56E-02 |
| GO:0005496 | steroid binding | MF | 5 | 2.53E-02 | 1.56E-02 |
| GO:0003682 | chromatin binding | MF | 21 | 2.63E-02 | 6.56E-02 |
| Note: CC, Cellular component; MF, Molecular function; m^6^A, N6-methyladenosine. | | | | | |

**Supplementary Table S4 |** Correlation analysis of m6A methyltransferases and lncRNAs.

| **m^6^A** | **LncRNA** | **gene name** | **PCC** |
| --- | --- | --- | --- |
| ALKBH5 | XR_001466981.2 | LOC107053589 | 0.84144 |
| ALKBH5 | XR_001469923.2 | TRNAU1AP | 0.84854 |
| ALKBH5 | XR_003073642.1 | EIF1B | 0.884177 |
| ALKBH5 | XR_003074580.1 | LOC112532161 | 0.893058 |
| ALKBH5 | XR_003074880.1 | LOC112532320 | 0.811529 |
| ALKBH5 | XR_003075342.1 | LOC112532491 | 0.817142 |
| ALKBH5 | XR_003076612.1 | LOC112532973 | -0.8904 |
| ALKBH5 | XR_003076614.1 | FYTTD1 | 0.917649 |
| FTO | CLOUDSEQ_00013882 | ELOC | 0.966922 |
| FTO | NR_004440.1 | ADARB1 | 0.834827 |
| FTO | NR_036657.2 | C14H16orf59 | 0.831189 |
| FTO | XR_001462089.2 | LOC107049737 | 0.948852 |
| FTO | XR_001462590.2 | ARGLU1 | 0.882018 |
| FTO | XR_001463804.2 | LOC107051860 | 0.954233 |
| FTO | XR_001464259.2 | CXorf40B | 0.828817 |
| FTO | XR_001466004.2 | TRMT11 | 0.927171 |
| FTO | XR_001467266.2 | LOC107053731 | 0.894295 |
| FTO | XR_001468768.2 | LOC107054477 | 0.831572 |
| FTO | XR_001468840.1 | LOC107054512 | 0.818803 |
| FTO | XR_003071323.1 | LOC112530084 | -0.81525 |
| FTO | XR_003072628.1 | IGBP1L | 0.825244 |
| FTO | XR_003073091.1 | LOC112531349 | 0.842441 |
| FTO | XR_003074229.1 | HMGN3 | 0.954086 |
| FTO | XR_003074761.1 | LOC107051757 | -0.89536 |
| FTO | XR_003074821.1 | LOC107052080 | 0.875324 |
| FTO | XR_003075460.1 | WDR20 | 0.887453 |
| FTO | XR_003076687.1 | WDR33 | 0.979382 |
| FTO | XR_003077619.1 | RAD50 | 0.882624 |
| FTO | XR_210638.3 | BOD1L1 | 0.87466 |
| HNRNPA2B1 | CLOUDSEQ_00060023 | - | 0.987533 |
| HNRNPA2B1 | XR_001464733.2 | RFK | -0.82716 |
| HNRNPA2B1 | XR_001466660.2 | PDHX | -0.88884 |
| HNRNPA2B1 | XR_001469739.2 | DHRS3 | -0.8233 |
| HNRNPA2B1 | XR_001470262.2 | LOC107055210 | -0.84805 |
| HNRNPA2B1 | XR_003073205.1 | LOC107057257 | -0.83266 |
| HNRNPA2B1 | XR_003073795.1 | LOC101749223 | -0.9767 |
| HNRNPA2B1 | XR_003074033.1 | LOC112531892 | 0.84142 |
| HNRNPA2B1 | XR_003074770.1 | LOC112532268 | -0.81738 |
| HNRNPA2B1 | XR_003075003.1 | LOC422757 | 0.969987 |
| HNRNPA2B1 | XR_003075391.1 | LOC107053405 | -0.83648 |
| HNRNPA2B1 | XR_003076030.1 | LOC107053866 | 0.856478 |
| HNRNPA2B1 | XR_003076260.1 | GALNT3 | 0.884072 |
| HNRNPA2B1 | XR_140261.4 | LOC100857928 | 0.876454 |
| IGF2BP2 | CLOUDSEQ_00001146 | RPS19BP1 | 0.854465 |
| IGF2BP2 | CLOUDSEQ_00040516 | LOC112533250 | 0.860518 |
| IGF2BP2 | CLOUDSEQ_00046804 | MICA | 0.86932 |
| IGF2BP2 | XR_001463388.2 | LOC107051670 | 0.872075 |
| IGF2BP2 | XR_003073205.1 | LOC107057257 | -0.85225 |
| IGF2BP2 | XR_003073280.1 | LOC426097 | 0.811453 |
| IGF2BP3 | CLOUDSEQ_00002202 | - | 0.82937 |
| IGF2BP3 | CLOUDSEQ_00041925 | TMA7 | 0.849899 |
| IGF2BP3 | CLOUDSEQ_00046843 | MOGL4 | 0.95596 |
| IGF2BP3 | CLOUDSEQ_00060023 | - | 0.915086 |
| IGF2BP3 | CLOUDSEQ_00065545 | - | 0.965424 |
| IGF2BP3 | NR_102417.2 | GPATCH11 | 0.899696 |
| IGF2BP3 | XR_001461846.2 | DSN1 | 0.899622 |
| IGF2BP3 | XR_001463967.2 | LOC101750669 | 0.899852 |
| IGF2BP3 | XR_001464579.2 | PSMD12 | -0.86931 |
| IGF2BP3 | XR_001465007.1 | HIBADH | -0.82608 |
| IGF2BP3 | XR_001467194.2 | CNNM2 | 0.899696 |
| IGF2BP3 | XR_001468037.2 | LOC107054091 | -0.90678 |
| IGF2BP3 | XR_001469222.2 | LOC107054694 | 0.899656 |
| IGF2BP3 | XR_001469476.2 | DHRS11 | 0.900115 |
| IGF2BP3 | XR_001469739.2 | DHRS3 | -0.97567 |
| IGF2BP3 | XR_001469910.2 | KHDRBS1 | 0.961108 |
| IGF2BP3 | XR_001470468.2 | LOC107055381 | 0.822248 |
| IGF2BP3 | XR_003071150.1 | LOC107053357 | 0.90948 |
| IGF2BP3 | XR_003071634.1 | FBXO40 | 0.889964 |
| IGF2BP3 | XR_003072639.1 | LOC112530919 | -0.84093 |
| IGF2BP3 | XR_003072877.1 | LOC112531203 | 0.95912 |
| IGF2BP3 | XR_003073795.1 | LOC101749223 | -0.87589 |
| IGF2BP3 | XR_003073945.1 | LOC112531857 | 0.885292 |
| IGF2BP3 | XR_003074033.1 | LOC112531892 | 0.953617 |
| IGF2BP3 | XR_003074170.1 | LOC112531971 | -0.82145 |
| IGF2BP3 | XR_003075216.1 | DNAJC24 | 0.879631 |
| IGF2BP3 | XR_003076030.1 | LOC107053866 | 0.837633 |
| IGF2BP3 | XR_003076260.1 | GALNT3 | 0.99652 |
| IGF2BP3 | XR_140261.4 | LOC100857928 | 0.864441 |
| METTL14 | CLOUDSEQ_00001146 | RPS19BP1 | 0.816034 |
| METTL14 | CLOUDSEQ_00013882 | ELOC | 0.81406 |
| METTL14 | CLOUDSEQ_00064175 | CACNG7 | 0.947323 |
| METTL14 | CLOUDSEQ_00066103 | FUS | 0.912086 |
| METTL14 | XR_001462583.2 | ARGLU1 | 0.856206 |
| METTL14 | XR_001462590.2 | ARGLU1 | 0.896312 |
| METTL14 | XR_001466660.2 | PDHX | -0.89384 |
| METTL14 | XR_001467276.2 | LOC107053735 | 0.930188 |
| METTL14 | XR_001467702.2 | LOC107053912 | -0.83791 |
| METTL14 | XR_001468435.2 | LOC107054293 | -0.94198 |
| METTL14 | XR_001468840.1 | LOC107054512 | 0.812738 |
| METTL14 | XR_001469826.2 | CHMP7 | 0.838531 |
| METTL14 | XR_001471439.2 | RC1IPL | 0.900693 |
| METTL14 | XR_003072639.1 | LOC112530919 | -0.8194 |
| METTL14 | XR_003074229.1 | HMGN3 | 0.8329 |
| METTL14 | XR_003074466.1 | LOC112532116 | -0.86162 |
| METTL14 | XR_003074761.1 | LOC107051757 | -0.8406 |
| METTL14 | XR_003074929.1 | MAP9 | 0.866518 |
| METTL14 | XR_003075003.1 | LOC422757 | 0.832005 |
| METTL14 | XR_003077115.1 | POLG | 0.868839 |
| METTL14 | XR_003077619.1 | RAD50 | 0.822514 |
| METTL14 | XR_212250.3 | LOC100859478 | -0.86156 |
| METTL16 | CLOUDSEQ_00001146 | RPS19BP1 | 0.90118 |
| METTL16 | CLOUDSEQ_00040516 | LOC112533250 | 0.811946 |
| METTL16 | CLOUDSEQ_00046804 | MICA | 0.86167 |
| METTL16 | CLOUDSEQ_00063777 | - | -0.81666 |
| METTL16 | XR_001465265.2 | LOC107052649 | -0.84678 |
| METTL16 | XR_001466660.2 | PDHX | -0.86021 |
| METTL16 | XR_001470262.2 | LOC107055210 | -0.88823 |
| METTL16 | XR_003073205.1 | LOC107057257 | -0.95478 |
| METTL16 | XR_003073665.1 | LOC112531736 | -0.81671 |
| METTL16 | XR_003073795.1 | LOC101749223 | -0.83975 |
| METTL16 | XR_003074770.1 | LOC112532268 | -0.88386 |
| METTL3 | XR_001464055.2 | B3GNT2 | 0.898416 |
| METTL3 | XR_001467587.1 | LOC107053854 | 0.8636 |
| METTL3 | XR_003072673.1 | LOC112530954 | 0.860458 |
| RBM15 | CLOUDSEQ_00002202 | - | 0.845408 |
| RBM15 | CLOUDSEQ_00002848 | - | 0.954126 |
| RBM15 | CLOUDSEQ_00013882 | ELOC | 0.85865 |
| RBM15 | CLOUDSEQ_00016700 | - | 0.863739 |
| RBM15 | CLOUDSEQ_00066103 | FUS | 0.975792 |
| RBM15 | XR_001462089.2 | LOC107049737 | 0.850665 |
| RBM15 | XR_001462583.2 | ARGLU1 | 0.996938 |
| RBM15 | XR_001462590.2 | ARGLU1 | 0.965146 |
| RBM15 | XR_001463418.1 | LOC107051690 | 0.889964 |
| RBM15 | XR_001463804.2 | LOC107051860 | 0.815656 |
| RBM15 | XR_001465404.2 | CHCHD7 | 0.889964 |
| RBM15 | XR_001465436.2 | LEMD3 | 0.86121 |
| RBM15 | XR_001466004.2 | TRMT11 | 0.903338 |
| RBM15 | XR_001466268.2 | LOC101748935 | 0.821164 |
| RBM15 | XR_001466827.2 | LOC107053517 | 0.938396 |
| RBM15 | XR_001467276.2 | LOC107053735 | 0.845692 |
| RBM15 | XR_001467449.2 | SF3B1 | 0.930796 |
| RBM15 | XR_001467702.2 | LOC107053912 | -0.91133 |
| RBM15 | XR_001468768.2 | LOC107054477 | 0.983449 |
| RBM15 | XR_001468840.1 | LOC107054512 | 0.954633 |
| RBM15 | XR_001469219.2 | LOC107054691 | 0.889964 |
| RBM15 | XR_001469498.2 | RBM39 | 0.934813 |
| RBM15 | XR_001469826.2 | CHMP7 | 0.973425 |
| RBM15 | XR_001469861.2 | LOC101749127 | -0.98338 |
| RBM15 | XR_001469923.2 | TRNAU1AP | 0.855 |
| RBM15 | XR_001470454.1 | LOC107055372 | 0.889964 |
| RBM15 | XR_001470509.2 | LOC107055439 | 0.889964 |
| RBM15 | XR_003071634.1 | FBXO40 | 0.856908 |
| RBM15 | XR_003071870.1 | TAF11 | 0.974493 |
| RBM15 | XR_003072108.1 | LOC107050662 | -0.88118 |
| RBM15 | XR_003072628.1 | IGBP1L | 0.957294 |
| RBM15 | XR_003072639.1 | LOC112530919 | -0.88832 |
| RBM15 | XR_003073081.1 | LOC112531334 | 0.811471 |
| RBM15 | XR_003073091.1 | LOC112531349 | 0.958552 |
| RBM15 | XR_003074170.1 | LOC112531971 | -0.83226 |
| RBM15 | XR_003074248.1 | LOC107053051 | 0.878888 |
| RBM15 | XR_003074466.1 | LOC112532116 | -0.82839 |
| RBM15 | XR_003074761.1 | LOC107051757 | -0.86156 |
| RBM15 | XR_003074880.1 | LOC112532320 | 0.871652 |
| RBM15 | XR_003074903.1 | LOC101751220 | 0.890231 |
| RBM15 | XR_003074929.1 | MAP9 | 0.850917 |
| RBM15 | XR_003075342.1 | LOC112532491 | 0.886143 |
| RBM15 | XR_003075460.1 | WDR20 | 0.968824 |
| RBM15 | XR_003075487.1 | LOC112532555 | 0.878969 |
| RBM15 | XR_003075514.1 | LOC107053396 | 0.889964 |
| RBM15 | XR_003075700.1 | LOC112532619 | 0.944798 |
| RBM15 | XR_003076030.1 | LOC107053866 | 0.824784 |
| RBM15 | XR_003076612.1 | LOC112532973 | -0.84404 |
| RBM15 | XR_003076687.1 | WDR33 | 0.856138 |
| RBM15 | XR_003076933.1 | PIAS1 | 0.975819 |
| RBM15 | XR_003077024.1 | FAN1 | 0.889964 |
| RBM15 | XR_003077115.1 | POLG | 0.985592 |
| RBM15 | XR_003077137.1 | CFDP1 | 0.830784 |
| RBM15 | XR_003077263.1 | LOC107054294 | 0.889964 |
| RBM15 | XR_003077619.1 | RAD50 | 0.841503 |
| RBM15 | XR_003077808.1 | LOC112533492 | 0.8522 |
| RBM15 | XR_003078022.1 | LOC107054691 | 0.889964 |
| RBM15 | XR_003078028.1 | LOC112533588 | 0.862659 |
| RBM15 | XR_212250.3 | LOC100859478 | -0.83662 |
| RBM15B | CLOUDSEQ_00002202 | - | 0.904906 |
| RBM15B | CLOUDSEQ_00002848 | - | 0.914902 |
| RBM15B | XR_001462583.2 | ARGLU1 | 0.836881 |
| RBM15B | XR_001462590.2 | ARGLU1 | 0.830757 |
| RBM15B | XR_001466827.2 | LOC107053517 | 0.82215 |
| RBM15B | XR_001467273.1 | LOC107053732 | 0.914321 |
| RBM15B | XR_001468768.2 | LOC107054477 | 0.837481 |
| RBM15B | XR_001468840.1 | LOC107054512 | 0.904552 |
| RBM15B | XR_001469498.2 | RBM39 | 0.974677 |
| RBM15B | XR_001469861.2 | LOC101749127 | -0.91722 |
| RBM15B | XR_001469910.2 | KHDRBS1 | 0.838483 |
| RBM15B | XR_001469923.2 | TRNAU1AP | 0.956688 |
| RBM15B | XR_003071634.1 | FBXO40 | 0.912223 |
| RBM15B | XR_003071870.1 | TAF11 | 0.883287 |
| RBM15B | XR_003072639.1 | LOC112530919 | -0.84981 |
| RBM15B | XR_003074170.1 | LOC112531971 | -0.94964 |
| RBM15B | XR_003074580.1 | LOC112532161 | 0.862341 |
| RBM15B | XR_003075460.1 | WDR20 | 0.860434 |
| RBM15B | XR_003075487.1 | LOC112532555 | 0.890795 |
| RBM15B | XR_003076030.1 | LOC107053866 | 0.83326 |
| RBM15B | XR_003076612.1 | LOC112532973 | -0.84526 |
| RBM15B | XR_003076933.1 | PIAS1 | 0.820771 |
| RBM15B | XR_003077619.1 | RAD50 | 0.841077 |
| RBM15B | XR_140261.4 | LOC100857928 | 0.910891 |
| RBMX | CLOUDSEQ_00002202 | - | 0.929435 |
| RBMX | CLOUDSEQ_00013882 | ELOC | 0.870224 |
| RBMX | CLOUDSEQ_00041925 | TMA7 | 0.854926 |
| RBMX | CLOUDSEQ_00046843 | MOGL4 | 0.872664 |
| RBMX | CLOUDSEQ_00060023 | - | 0.883548 |
| RBMX | CLOUDSEQ_00065545 | - | 0.933366 |
| RBMX | CLOUDSEQ_00066103 | FUS | 0.842605 |
| RBMX | XR_001462590.2 | ARGLU1 | 0.876586 |
| RBMX | XR_001466660.2 | PDHX | -0.8228 |
| RBMX | XR_001468840.1 | LOC107054512 | 0.917812 |
| RBMX | XR_001469498.2 | RBM39 | 0.815602 |
| RBMX | XR_001469739.2 | DHRS3 | -0.84564 |
| RBMX | XR_001469910.2 | KHDRBS1 | 0.949739 |
| RBMX | XR_003071150.1 | LOC107053357 | 0.972093 |
| RBMX | XR_003071634.1 | FBXO40 | 0.970932 |
| RBMX | XR_003072628.1 | IGBP1L | 0.833978 |
| RBMX | XR_003072639.1 | LOC112530919 | -0.96322 |
| RBMX | XR_003072877.1 | LOC112531203 | 0.813672 |
| RBMX | XR_003074033.1 | LOC112531892 | 0.873026 |
| RBMX | XR_003074170.1 | LOC112531971 | -0.88376 |
| RBMX | XR_003075003.1 | LOC422757 | 0.825856 |
| RBMX | XR_003076030.1 | LOC107053866 | 0.867629 |
| RBMX | XR_003076260.1 | GALNT3 | 0.897722 |
| RBMX | XR_003076687.1 | WDR33 | 0.841919 |
| RBMX | XR_003076933.1 | PIAS1 | 0.819828 |
| RBMX | XR_003077619.1 | RAD50 | 0.93673 |
| RBMX | XR_140261.4 | LOC100857928 | 0.896534 |
| RBMX | XR_210638.3 | BOD1L1 | 0.883433 |
| WTAP | CLOUDSEQ_00002202 | - | 0.883507 |
| WTAP | CLOUDSEQ_00013882 | ELOC | 0.925826 |
| WTAP | CLOUDSEQ_00023400 | LOC107053245 | 0.95521 |
| WTAP | CLOUDSEQ_00041925 | TMA7 | 0.928326 |
| WTAP | CLOUDSEQ_00065545 | - | 0.871763 |
| WTAP | CLOUDSEQ_00065872 | LOC107049904 | 0.82697 |
| WTAP | XR_001468840.1 | LOC107054512 | 0.816746 |
| WTAP | XR_001469910.2 | KHDRBS1 | 0.8126 |
| WTAP | XR_003071150.1 | LOC107053357 | 0.902971 |
| WTAP | XR_003071634.1 | FBXO40 | 0.815613 |
| WTAP | XR_003072639.1 | LOC112530919 | -0.87939 |
| WTAP | XR_003074786.1 | LOC112532277 | -0.83914 |
| WTAP | XR_003076687.1 | WDR33 | 0.917976 |
| WTAP | XR_003077619.1 | RAD50 | 0.865163 |
| WTAP | XR_210638.3 | BOD1L1 | 0.821993 |
| YTHDC1 | CLOUDSEQ_00002202 | - | 0.824286 |
| YTHDC1 | CLOUDSEQ_00002848 | - | 0.881661 |
| YTHDC1 | CLOUDSEQ_00013882 | ELOC | 0.920715 |
| YTHDC1 | CLOUDSEQ_00018352 | MAP3K5 | -0.81246 |
| YTHDC1 | CLOUDSEQ_00066103 | FUS | 0.932199 |
| YTHDC1 | NR_004440.1 | ADARB1 | 0.818213 |
| YTHDC1 | XR_001462089.2 | LOC107049737 | 0.938541 |
| YTHDC1 | XR_001462583.2 | ARGLU1 | 0.967453 |
| YTHDC1 | XR_001462590.2 | ARGLU1 | 0.971971 |
| YTHDC1 | XR_001463418.1 | LOC107051690 | 0.860087 |
| YTHDC1 | XR_001463804.2 | LOC107051860 | 0.92105 |
| YTHDC1 | XR_001463998.1 | C1H2ORF49 | 0.8306 |
| YTHDC1 | XR_001465404.2 | CHCHD7 | 0.860087 |
| YTHDC1 | XR_001466004.2 | TRMT11 | 0.971378 |
| YTHDC1 | XR_001466827.2 | LOC107053517 | 0.836348 |
| YTHDC1 | XR_001467266.2 | LOC107053731 | 0.875824 |
| YTHDC1 | XR_001467276.2 | LOC107053735 | 0.824374 |
| YTHDC1 | XR_001467449.2 | SF3B1 | 0.869659 |
| YTHDC1 | XR_001467702.2 | LOC107053912 | -0.90973 |
| YTHDC1 | XR_001468768.2 | LOC107054477 | 0.983002 |
| YTHDC1 | XR_001468840.1 | LOC107054512 | 0.920158 |
| YTHDC1 | XR_001469219.2 | LOC107054691 | 0.860087 |
| YTHDC1 | XR_001469498.2 | RBM39 | 0.87298 |
| YTHDC1 | XR_001469826.2 | CHMP7 | 0.900493 |
| YTHDC1 | XR_001469861.2 | LOC101749127 | -0.9568 |
| YTHDC1 | XR_001469923.2 | TRNAU1AP | 0.819761 |
| YTHDC1 | XR_001469926.2 | LOC107055019 | 0.835344 |
| YTHDC1 | XR_001470454.1 | LOC107055372 | 0.860087 |
| YTHDC1 | XR_001470509.2 | LOC107055439 | 0.860087 |
| YTHDC1 | XR_003071870.1 | TAF11 | 0.961699 |
| YTHDC1 | XR_003072108.1 | LOC107050662 | -0.88561 |
| YTHDC1 | XR_003072628.1 | IGBP1L | 0.935077 |
| YTHDC1 | XR_003072639.1 | LOC112530919 | -0.85162 |
| YTHDC1 | XR_003073081.1 | LOC112531334 | 0.874018 |
| YTHDC1 | XR_003073091.1 | LOC112531349 | 0.970091 |
| YTHDC1 | XR_003074229.1 | HMGN3 | 0.887696 |
| YTHDC1 | XR_003074248.1 | LOC107053051 | 0.831044 |
| YTHDC1 | XR_003074761.1 | LOC107051757 | -0.91354 |
| YTHDC1 | XR_003074903.1 | LOC101751220 | 0.863035 |
| YTHDC1 | XR_003075342.1 | LOC112532491 | 0.850508 |
| YTHDC1 | XR_003075460.1 | WDR20 | 0.990121 |
| YTHDC1 | XR_003075514.1 | LOC107053396 | 0.860087 |
| YTHDC1 | XR_003075700.1 | LOC112532619 | 0.940304 |
| YTHDC1 | XR_003076687.1 | WDR33 | 0.929365 |
| YTHDC1 | XR_003076933.1 | PIAS1 | 0.934654 |
| YTHDC1 | XR_003077024.1 | FAN1 | 0.860087 |
| YTHDC1 | XR_003077115.1 | POLG | 0.962017 |
| YTHDC1 | XR_003077263.1 | LOC107054294 | 0.860087 |
| YTHDC1 | XR_003077619.1 | RAD50 | 0.86786 |
| YTHDC1 | XR_003077808.1 | LOC112533492 | 0.888656 |
| YTHDC1 | XR_003078022.1 | LOC107054691 | 0.860087 |
| YTHDC1 | XR_003078028.1 | LOC112533588 | 0.844229 |
| YTHDC1 | XR_210638.3 | BOD1L1 | 0.819871 |
| YTHDC2 | CLOUDSEQ_00002202 | - | 0.940737 |
| YTHDC2 | CLOUDSEQ_00002848 | - | 0.929936 |
| YTHDC2 | CLOUDSEQ_00013882 | ELOC | 0.926408 |
| YTHDC2 | CLOUDSEQ_00065872 | LOC107049904 | 0.834927 |
| YTHDC2 | CLOUDSEQ_00066103 | FUS | 0.862056 |
| YTHDC2 | XR_001462583.2 | ARGLU1 | 0.875306 |
| YTHDC2 | XR_001462590.2 | ARGLU1 | 0.875514 |
| YTHDC2 | XR_001463804.2 | LOC107051860 | 0.814564 |
| YTHDC2 | XR_001465436.2 | LEMD3 | 0.947831 |
| YTHDC2 | XR_001466004.2 | TRMT11 | 0.878138 |
| YTHDC2 | XR_001466827.2 | LOC107053517 | 0.887191 |
| YTHDC2 | XR_001467449.2 | SF3B1 | 0.885929 |
| YTHDC2 | XR_001468768.2 | LOC107054477 | 0.888349 |
| YTHDC2 | XR_001468840.1 | LOC107054512 | 0.960535 |
| YTHDC2 | XR_001469861.2 | LOC101749127 | -0.84778 |
| YTHDC2 | XR_001469910.2 | KHDRBS1 | 0.853351 |
| YTHDC2 | XR_003071150.1 | LOC107053357 | 0.845175 |
| YTHDC2 | XR_003071634.1 | FBXO40 | 0.888675 |
| YTHDC2 | XR_003071870.1 | TAF11 | 0.870403 |
| YTHDC2 | XR_003072108.1 | LOC107050662 | -0.83618 |
| YTHDC2 | XR_003072430.1 | CCL28 | 0.866241 |
| YTHDC2 | XR_003072628.1 | IGBP1L | 0.968301 |
| YTHDC2 | XR_003072639.1 | LOC112530919 | -0.96403 |
| YTHDC2 | XR_003073091.1 | LOC112531349 | 0.903743 |
| YTHDC2 | XR_003074170.1 | LOC112531971 | -0.91135 |
| YTHDC2 | XR_003074761.1 | LOC107051757 | -0.85226 |
| YTHDC2 | XR_003074786.1 | LOC112532277 | -0.9389 |
| YTHDC2 | XR_003075460.1 | WDR20 | 0.884411 |
| YTHDC2 | XR_003075487.1 | LOC112532555 | 0.888051 |
| YTHDC2 | XR_003076030.1 | LOC107053866 | 0.818291 |
| YTHDC2 | XR_003076687.1 | WDR33 | 0.918873 |
| YTHDC2 | XR_003076933.1 | PIAS1 | 0.947879 |
| YTHDC2 | XR_003077115.1 | POLG | 0.817447 |
| YTHDC2 | XR_003077137.1 | CFDP1 | 0.900338 |
| YTHDC2 | XR_003077619.1 | RAD50 | 0.85193 |
| YTHDF1 | CLOUDSEQ_00002202 | - | 0.859233 |
| YTHDF1 | CLOUDSEQ_00002848 | - | 0.836346 |
| YTHDF1 | CLOUDSEQ_00060023 | - | 0.895212 |
| YTHDF1 | XR_001467273.1 | LOC107053732 | 0.928063 |
| YTHDF1 | XR_001468840.1 | LOC107054512 | 0.859817 |
| YTHDF1 | XR_001469498.2 | RBM39 | 0.94608 |
| YTHDF1 | XR_001469861.2 | LOC101749127 | -0.85479 |
| YTHDF1 | XR_001469910.2 | KHDRBS1 | 0.876385 |
| YTHDF1 | XR_001469923.2 | TRNAU1AP | 0.851338 |
| YTHDF1 | XR_003071150.1 | LOC107053357 | 0.820688 |
| YTHDF1 | XR_003071634.1 | FBXO40 | 0.925966 |
| YTHDF1 | XR_003072639.1 | LOC112530919 | -0.83246 |
| YTHDF1 | XR_003073795.1 | LOC101749223 | -0.82846 |
| YTHDF1 | XR_003074170.1 | LOC112531971 | -0.90522 |
| YTHDF1 | XR_003074580.1 | LOC112532161 | 0.815975 |
| YTHDF1 | XR_003075003.1 | LOC422757 | 0.922129 |
| YTHDF1 | XR_003075487.1 | LOC112532555 | 0.840492 |
| YTHDF1 | XR_003076030.1 | LOC107053866 | 0.888962 |
| YTHDF1 | XR_003076260.1 | GALNT3 | 0.821296 |
| YTHDF1 | XR_003077619.1 | RAD50 | 0.823554 |
| YTHDF1 | XR_140261.4 | LOC100857928 | 0.963064 |
| YTHDF2 | CLOUDSEQ_00046804 | MICA | 0.825734 |
| YTHDF2 | CLOUDSEQ_00063777 | - | -0.91085 |
| YTHDF2 | XR_001467273.1 | LOC107053732 | 0.849935 |
| YTHDF2 | XR_001469498.2 | RBM39 | 0.928207 |
| YTHDF2 | XR_001469861.2 | LOC101749127 | -0.86367 |
| YTHDF2 | XR_001469923.2 | TRNAU1AP | 0.898005 |
| YTHDF2 | XR_003074580.1 | LOC112532161 | 0.973041 |
| YTHDF2 | XR_003075003.1 | LOC422757 | 0.84105 |
| YTHDF2 | XR_003076612.1 | LOC112532973 | -0.90429 |
| YTHDF2 | XR_003078028.1 | LOC112533588 | 0.838625 |
| YTHDF2 | XR_140261.4 | LOC100857928 | 0.823235 |
| YTHDF3 | CLOUDSEQ_00001146 | RPS19BP1 | 0.883953 |
| YTHDF3 | CLOUDSEQ_00063777 | - | -0.93243 |
| YTHDF3 | XR_001463998.1 | C1H2ORF49 | 0.895973 |
| YTHDF3 | XR_001466660.2 | PDHX | -0.81178 |
| YTHDF3 | XR_001467276.2 | LOC107053735 | 0.881612 |
| YTHDF3 | XR_001470262.2 | LOC107055210 | -0.82878 |
| YTHDF3 | XR_003073205.1 | LOC107057257 | -0.84504 |
| YTHDF3 | XR_003074580.1 | LOC112532161 | 0.813752 |
| YTHDF3 | XR_003074770.1 | LOC112532268 | -0.82216 |
| YTHDF3 | XR_003078028.1 | LOC112533588 | 0.867963 |
| ZC3H13 | CLOUDSEQ_00001146 | RPS19BP1 | 0.867969 |
| ZC3H13 | CLOUDSEQ_00002202 | - | 0.931863 |
| ZC3H13 | CLOUDSEQ_00041925 | TMA7 | 0.851423 |
| ZC3H13 | CLOUDSEQ_00046843 | MOGL4 | 0.849994 |
| ZC3H13 | CLOUDSEQ_00060023 | - | 0.828533 |
| ZC3H13 | CLOUDSEQ_00065545 | - | 0.877074 |
| ZC3H13 | XR_001462089.2 | LOC107049737 | 0.832962 |
| ZC3H13 | XR_001462590.2 | ARGLU1 | 0.854394 |
| ZC3H13 | XR_001466660.2 | PDHX | -0.81402 |
| ZC3H13 | XR_001467266.2 | LOC107053731 | 0.883933 |
| ZC3H13 | XR_001467276.2 | LOC107053735 | 0.822331 |
| ZC3H13 | XR_001467790.2 | LRRC8C | -0.81834 |
| ZC3H13 | XR_001468840.1 | LOC107054512 | 0.879237 |
| ZC3H13 | XR_001469498.2 | RBM39 | 0.895035 |
| ZC3H13 | XR_001469861.2 | LOC101749127 | -0.8356 |
| ZC3H13 | XR_001469910.2 | KHDRBS1 | 0.895127 |
| ZC3H13 | XR_003071150.1 | LOC107053357 | 0.955644 |
| ZC3H13 | XR_003071634.1 | FBXO40 | 0.958914 |
| ZC3H13 | XR_003072639.1 | LOC112530919 | -0.8794 |
| ZC3H13 | XR_003072714.1 | LOC112530996 | 0.889282 |
| ZC3H13 | XR_003074170.1 | LOC112531971 | -0.91217 |
| ZC3H13 | XR_003075003.1 | LOC422757 | 0.811645 |
| ZC3H13 | XR_003075460.1 | WDR20 | 0.81571 |
| ZC3H13 | XR_003076260.1 | GALNT3 | 0.821176 |
| ZC3H13 | XR_003077619.1 | RAD50 | 0.96394 |
| ZC3H13 | XR_140261.4 | LOC100857928 | 0.980829 |
| ZC3H13 | XR_210638.3 | BOD1L1 | 0.950061 |
| Note: m^6^A, N6-methyladenosine; PCC, Pearson correlation coefficient. | | | |
